# Respiration kinetics and allometric scaling in the demosponge *Halichondria panicea*

**DOI:** 10.1186/s12862-023-02163-5

**Published:** 2023-09-19

**Authors:** Lars Kumala, Malte Thomsen, Donald E. Canfield

**Affiliations:** 1 Department of Biology, University of Southern Denmark, Odense M, 5230 Denmark; 2 Marine Biological Research Centre, University of Southern Denmark, Kerteminde, 5300 Denmark; 3 Nordcee, Department of Biology, University of Southern Denmark, Odense M, 5230 Denmark; 4 Danish Institute for Advanced Study (DIAS), University of Southern Denmark, Odense M, 5230 Denmark

**Keywords:** Oxygen, Respiration kinetics, Sponges, Modularity, Metabolic scaling, Animal evolution

## Abstract

**Background:**

The aquiferous system in sponges represents one of the simplest circulatory systems used by animals for the internal uptake and distribution of oxygen and metabolic substrates. Its modular organization enables sponges to metabolically scale with size differently than animals with an internal circulatory system. In this case, metabolic rate is typically limited by surface to volume constraints to maintain an efficient supply of oxygen and food. Here, we consider the linkeage between oxygen concentration, the respiration rates of sponges and sponge size.

**Results:**

We explored respiration kinetics for individuals of the demosponge *Halichondria panicea* with varying numbers of aquiferous modules (*n*_*modules*_ = 1–102). From this work we establish relationships between the sponge size, module number, maximum respiration rate (*R*_*max*_) and the half-saturation constant, *K*_*m*_, which is the oxygen concentration producing half of the maximum respiration rate, *R*_*max*_. We found that the *n*_*modules*_ in *H. panicea* scales consistently with sponge volume (*V*_*sp*_) and that *R*_*max*_ increased with sponge size with a proportionality > 1. Conversly, we found a lack of correlation between *K*_*m*_ and sponge body size suggesting that oxygen concentration does not control the size of sponges.

**Conclusions:**

The present study reveals that the addition of aquiferous modules (with a mean volume of 1.59 ± 0.22 mL) enables *H. panicea* in particular, and likely demosponges in general, to grow far beyond constraints limiting the size of their component modules and independent of ambient oxygen levels.

**Supplementary Information:**

The online version contains supplementary material available at 10.1186/s12862-023-02163-5.

## Background

Most aerobic, unicellular organisms that rely on molecular diffusion for oxygen uptake and intracellular transport cannot exceed a diameter of 1–2 mm since beyond this threshold, oxygen supply cannot be maintained by diffusion alone [1]. To evolve into larger organisms, more sophisticated systems for oxygen delivery and distribution had to be developed. The development of circulatory systems including respiratory pigments (e.g. haemoglobin, haemocyanin) for the internal, convective, transport of oxygen enables multicellular organisms to overcome physical limitations of diffusive oxygen supply associated with an increase in size. Such limitations include an extended pathway length for diffusion and a decrease in the surface to volume ratio [1]. However, oxygen may still dictate the size limit of an aerobic, multicellular organism since its net transport within large animals, with morphologically more complex systems, necessitates high ambient oxygen levels [2]. One of the most primitive, but yet highly efficient, circulatory systems used by animals is found in sponges (Porifera), who probably represent the earliest-branching multicellular lineage of extant animals [3–7].

The supply of oxygen and food to the sponge interior is facilitated through the convective transport of ambient seawater in the internal water canal system. In leuconoid demosponges, such as *Halichondria panicea*, seawater is impelled by the beating action of flagellated choanocytes, which are organized in multiple internal chambers (choanocyte chambers) to overcome the pressure resistance of the extensive water canal system [8–10]. Water enters the sponge through inhalant openings (ostia) in the surface (exopinacoderm) and flows internally through a water canal system merging into one or several exhalant opening(s) (osculum/oscula). This internal water flow facilitates highly efficient filtering of a substantial volume of water [11], supplies the sponge interior with oxygen and removes waste products [12–15].

Although sponges lack nerves and true muscles [16, 17], they can modulate hydrodynamic pressure differentials, and thus the internal water flow. They do this through contraction–expansion of the aquiferous system including the incurrent pores and the exhalant opening(s) [8, 9, 18]. Contractile behaviour is coordinated by actin microfilaments, myocytes and actinocytes lining the different compartments of the water canal system [19–21] and contractions temporary limit the internal volumetric flow that supplies the sponge interior with food as well as oxygen [22, 23].

The aquiferous system in *Halichondria panicea* is subdivided into repeated, structural–functional units known as aquiferous modules, each with it’s own osculum. Each module corresponds to a specific volume in the sponge that is supplied by a system of choanocyte chambers and water canals [24, 25]. These modules are physically integrated to varying degrees into a structural individual [24, 25] as they sometimes share an exopinacoderm and/or common mesohyl where sponge cells (e.g. amoebocytes) can migrate freely [12, 13, 26]. Depending on the size of the sponge, several aquiferous modules may be present in an individual [25]. This modular organization enables sponges to attain enormous sizes [27] and to form remarkably stable populations since it empowers large specimens to endure partial mortality caused by, for example, predation or detrimental environmental conditions [28–30]. Such modular design may also permit sponges to metabolically scale differently with increasing size compared to unitary organisms, i.e. organisms with a distinct developmental plan as in most animals. The cumulative formation of aquiferous modules during growth may only have minimal impact on the pre-existing modules if the ‘new’ units provide for themselves with respect to food and oxygen uptake as well as waste management.

The relationship between the metabolic rate of an organism and its size is typically given by the power function [31, 32]:

1 $$Y={aM}^{b}$$

here, the power exponent *b* defines the scaling of metabolic (i.e. aerobic respiration) rate, *Y,* with the organism mass, *M*, given by the scaling coefficient *a*. Unitary organisms, such as mammals, birds or insects, frequently show a scaling exponent ranging from ~ 2/3 to ~ 3/4 [33–35], which is typically known as Kleiber’s law [32, 34, 36]. Metabolic allometric relationships with *b* < 1 (i.e. *b* = 2/3 or ¾) imply that larger specimens have a lower metabolic rate per unit mass than their smaller counterparts. The most common mechanistic theory to explain such metabolic allometry is that the volume of an organism increases faster during growth than the surface area across which metabolic substrates and oxygen are exchanged between the external and internal environment [37, 38]. This change in the surface area-to-volume ratio confines the flux of nutrients, oxygen and/or waste [37, 39].

However, the actual mechanisms underpinning the “¾-power law” are unclear, in part because large differences in the metabolic scaling exponent can be observed both within and between species [39–42]. Still, this observed range of patterns in metabolic scaling is usually linked to either variations in the fractal geometry of resource-transport networks [43], or to surface area, mass, as well as energetic constraints (e.g. see reviews by [37, 38, 44]).

Modular organisms, such as bryozoans [45], or perhaps sponges [46], may represent an exception to the metabolic allometry associated with Kleiber’s law due to their ability to proportionally enlarge the interface between the external and internal environment by simply adding (aquiferous) modules during growth. The addition of similar, independent, modules permits the maintainance of an optimal surface-to-volume ratio (i.e. *SV*-ratio) for respiration, resource gathering and distribution [42, 45], independent of organism size. Thus, a sponge can change its whole-organism biomass without changing its mass-specific metabolic rate (i.e. *b* = 1; [46, 47]).

**Fig. 1:**
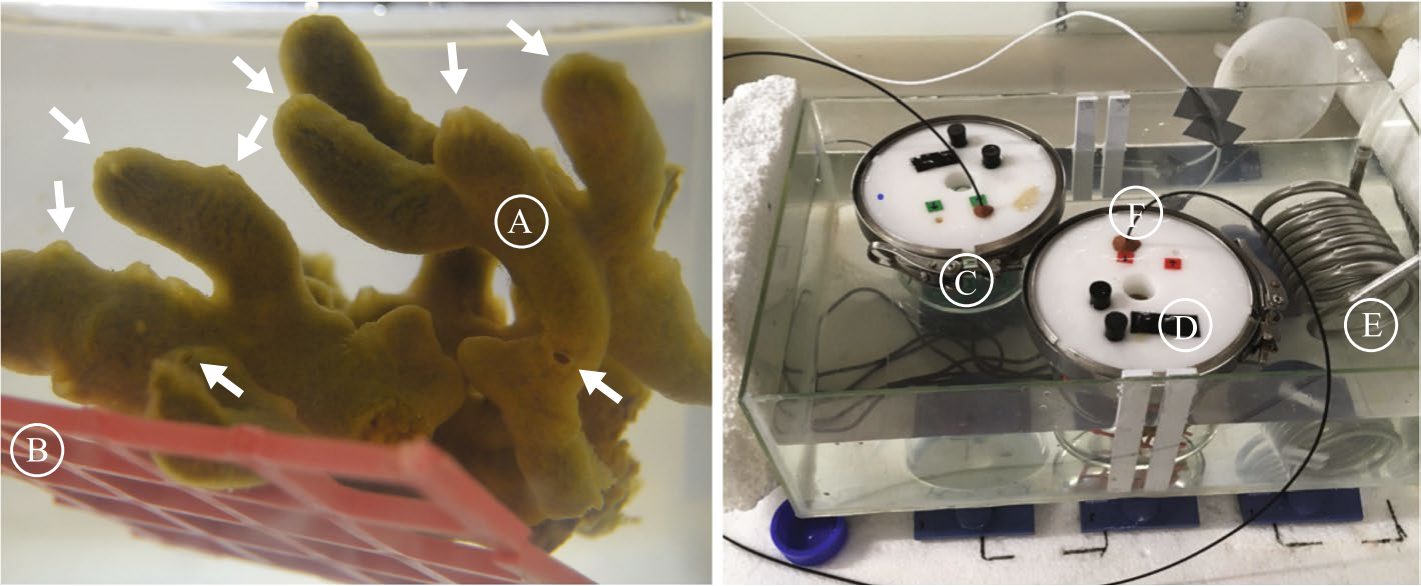
Left panel: Multi-oscula *Halichondria panicea* specimen (**A**) on a PVC net (**B**) in respiration chamber with well-mixed seawater (*T* = 12.4 ± 0.1 °C, *S* = 20 PSU). Each osculum (indicated by the white arrow) corresponds to an aquiferous module, representing a specific volume in the sponge that is supplied by an internal system of water canals [24, 25]. Right panel: Respiration chambers with (**C**) and without sponge (= control, **D**) were submerged in a temperature-controlled water-bath (**E**) to minimize temperature variations during measurements of the dissolved oxygen (*DO*, % AS) in respiration chambers. Optical fibers were attached to a contactless oxygen sensor spot glued to the inner wall of the chamber lid (**F**) to monitor the *DO* using FireStingO_2_ optical oxygen meter (PyroScience, Germany)

In this study, we explored the respiration kinetics of *H. panicea* of varying size to elucidate the linkeage between the metabolic (i.e. respiration) rate, sponge size as well as their oxygen dependencies. First, we performed oxygen drawdown experiments with *H. panicea* sponges possessing numerous aquiferous modules (*n*_*modules*_ = 1 – 102, Fig. 1) to determine their respiration rate under a wide range of oxygen levels. We then established relationships between sponge size (i.e. number of modules, dry weight, volume, module volume and spherical diameter) and the maximum respiration rate (*R*_*max*_) as well as the half-saturation constant (*K*_*m*_) of oxygen concentration during oxygen respiration. The *K*_*m*_ represents the oxygen level that supports half of the *R*_*max*_ [2, 48]. The relationship between the sponge size and the *K*_*m*_ may reveal how the modular-designed aquiferous system in sponges could impact sponge size at low-oxygen concentrations.

## Results

### Oxygen drawdown by sponges

**Fig. 2:**
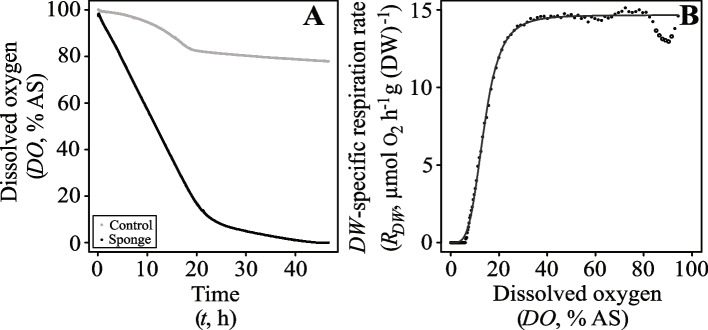
**A** Example of oxygen (% AS) drawdown recorded in respiration chamber with (*ID*1, dark line) and without a sponge (i.e. control, grey line). **B** Dry weight-specific respiration rate (*R*_*DW*_, µmol O_2_ h^−1^ g (DW)^−1^) of a sponge (*ID*1) plotted as a function of dissolved oxygen (*DO,* % AS) in seawater. Open circles indicate outliers which were not considered for determinations of the maximum *R*_*DW*_ and the half-saturation constant *K*_*m*_ (% AS) (Additional file 1: Figures S1, S2). Line represents model fit

During our respiration experiments, we observed a rapid, initial draw-down of dissolved oxygen (*DO*) in the respiration chambers that slowed as the *DO* reached lower concentrations (example in Fig. 2A). Overall, we measured a nearly constant dry-weight specific respiration rate (*R*_*DW*,_ µmol O_2_ h^−1^ g (DW)^−1^) of *H. panicea* sponges (*n* = 13) with *DO* > 20% AS, followed by a sudden decrease and subsequent diminished *R*_*DW*_ as the oxygen levels in the respiration chamber approached anoxia (Fig. 2B). The relationship between the *R*_*DW*_ and the ambient oxygen level (*DO*, % air saturation) was best described using the Hill equation (Eq. 4) as supported by a strong model fit (*R*^*2*^ > 0.98) (Fig. 2B and Additional file 1: Figure S2). The shape of the saturation curve varied between sigmoidal for the smallest specimens to nearly hyperbolic, as shown by the Hill coefficient ranging from 4.40 (*ID*2) to 1.10 (*ID*10) (Additional file 1: Figure S2).

Both the half-saturation constant (*K*_*m*_, % AS) and the maximal *R*_*DW*_ (*R*_*max*_*,* µmol O_2_ h^−1^ g (DW)^−1^) determined by the Hill model varied between individual sponges, with *K*_*m*_ ranging between 4.22 – 28.83% AS and *R*_*max*_ ranging between 6.88 – 33.79 µmol O_2_ h^−1^ g (DW)^−1^. The mean *K*_*m*_ (± 95% confidence intervals) and the mean *R*_*max*_ (± CI_95%_) for all specimens was 12.74 ± 4.14% AS and 19.00 ± 4.79 µmol O_2_ h^−1^ g (DW)^−1^, respectively (Table 1).

**Table 1:** Number of modules, (*n*_*modules)*_, sponge volume (*V*_*sp*_), module volume (*V*_*module*_), spherical diameter (*d*) and dry-weight (*DW*) of several *H. panicea* sponges (*ID*1-13) used in oxygen drawdown experiments to determine their half-saturation constant (*K*_*m*_) and their maximum dry weight-specific respiration (*R*_*max*_). Mean and 95% confidence intervals (CI_95%_) are shown

*ID*	*n*_*modules*_	*V*_*sp*_ (mL)	*V*_*module*_ (mL)	*d* (cm)	*DW* (g)	*K*_*m*_ (% AS)	*R*_*max*_ (µmol h^−1^ g (DW)^−1^)
1	1	1.30	1.30	1.35	0.08	13.77	14.67
2	2	4.10	2.05	1.99	0.16	6.15	13.49
3	2	2.95	1.48	1.78	0.18	8.20	13.58
4	2	3.75	1.88	1.93	0.21	15.82	9.21
5	NA	3.85	NA	1.94	0.28	23.26	8.98
6	4	7.00	1.75	2.37	0.49	11.63	6.88
7	21	29.00	1.38	3.81	1.12	8.20	21.80
8	58	71.00	1.22	5.14	3.39	11.44	20.58
9	102	127.60	1.25	6.25	10.74	5.82	30.70
10	47	51.30	1.09	4.61	2.83	21.39	33.79
11	14	19.00	1.36	3.31	0.98	4.22	30.45
12	41	80.00	1.95	5.35	6.55	28.83	21.72
13	72	170.00	2.36	6.87	19.44	6.89	21.11
Mean ± CI_95%_			1.59 ± 0.22			12.74 ± 4.14	19.00 ± 4.79

### Scaling relationships in multi-oscula *Halichondria panicea* sponges

**Fig. 3:**
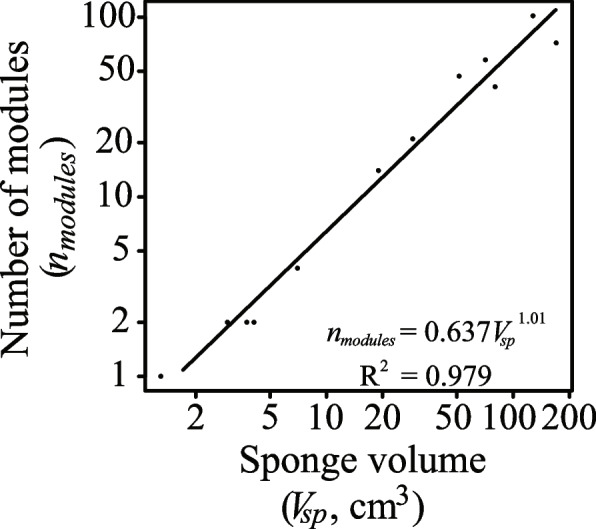
Relationship between the number of modules (*n*_*modules*_) and sponge volume (*V*_*sp*_, cm^3^) plotted on a double logarithmic scale

We investigated the relationship between the number of aquiferous modules (i.e. oscula; *n*_*modules*_), and sponge size in our *H. panicea* specimens (*n* = 13). The *n*_*modules*_ ranged between 1 to 102, and the mean volume of modules (*V*_*module*_ ± 95% confidence intervals) in our *H. panicea* sponges was 1.59 ± 0.22 mL (*n* = 12, Table 1). We found that the relationship between *n*_*modules*_ and the sponge volume (*V*_*sp*_, mL) of *H. panicea* sponges can be expressed by the equation *n*_*modules*_ = 0.637 × *V*_*sp*_^1.01^ (Fig. 3), essentially a linear response*.*

**Fig. 4:**
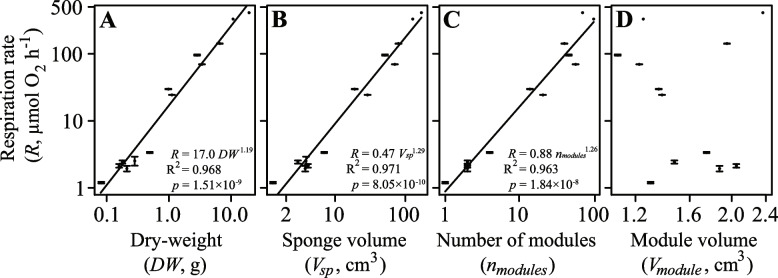
Maximum respiration rate (*R*, µmol O_2_ h^−1^) as a function of **A**) the sponge dry weight (*DW*, g), **B**) volume (*V*_*sp*_, cm^3^), **C**) number of modules (*n*_*modules*_) and **D**) module volume (*V*_*module*_, cm^3^) plotted on a double logarithmic scale. Regression line (dark line) and equation of the fitted power function is shown

We further explored scaling of the metabolic rate, *R* (µmol O_2_ h^−1^), with sponge biomass (*DW*, g), volume (*V*_*sp*_, mL) as well as *n*_*modules*_ in these sponges. We found that the relationship between the *R* and *DW* was described as *R* = 17.0 × *DW*^1.19^ (Fig. 4 A) whereas the relationship between *R* and *V*_*sp*_ was *R* = 0.47 × *V*_*sp*_^1.29^(Fig. 4 B). Our scaling exponents of *b* = 1.19 (*t* = -2.381, df = 22, *p* = 0.026) and 1.29 (*t* = -3.917, df = 22, *p* = 7.38 × 10^–4^) were significantly different from isometric scaling (*b* = 1). The scaling between the *R* and the *n*_*modules*_ can be described as *R* = 0.88 × *n*_*modules*_^1.26^ (Fig. 4 C). The scaling exponent of *β* = 1.26 was significantly different from *b* = 1 (*t* = -2.817, *df* = 20, *p* = 0.011). We found no correlation (*R*^2^ = 0.063) between the *R* and the module volume (*V*_*module*_, cm^3^) (Fig. 4 D).

**Fig. 5:**
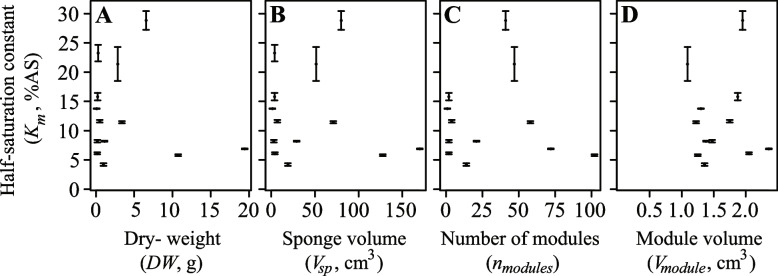
Half-saturation constant (*K*_*m*_, % AS) as a function of sponge size, i.e. **A**) sponge dry weight (*DW*, g), **B**) sponge volume (*V*_*sp*_, cm^3^), **C**) number of modules (*n*_*modules*_), and **D**) module volume (*V*_*module*_, cm^3^). Error bars represent the standard error (SE) of the model fit

We also investigated scaling of the half-saturation constant (*K*_*m*_, % AS) with sponge *DW*, *V*_*sp*_, *n*_*modules*_ and *V*_*module*_. However, these relationships could not be described by an allometric function, as *K*_*m*_ did not correlate significantly with any of these parameters [i.e. *DW* (*R*^2^ = 0.005), *V*_*sp*_ (*R*^2^ = 0.012), *n*_*modules*_ (*R*^2^ = 4.43 × 10^–5^) and *V*_*module*_ (*R*^2^ = 5 × 10^–4^)] (Fig. 5).

## Discussion

### Scaling of sponge respiration rate with size

As noted in the Introduction, the respiration rate *R* of an organism is closely related to its body size (*M*) as typically expressed through Eq. 1, and when *b* =  ~ ¾, the expression is known as Kleiber’s law [32]. As also noted, a *b* exponent of < 1 implies that larger specimens of a species have lower metabolic rates per unit mass (or size) than their smaller counterparts.

The present study reinforces the idea that sponges deviate from Kleiber’s law [46, 47, 49]. We show that the respiration rate (*R*, µmol O_2_ h^−1^) of *H. panicea* scales with sponge dry weight (*DW*) in a power function with an exponent of *b* = 1.19 (Fig. 4A) and respiration rate scales with volume (*V*_*sp*_) with *b* = 1.29 (Fig. 4B). These *b* exponents are greater than 1, and thus greater than we originally hypothesized. These *b* exponents also imply that larger sponges have greater weight- and volume-specific respiration rates compared to smaller sponges.

This finding of positive allometry, i.e. *b* > 1, is unusual and would seem to contrast with the metabolic isometry found in previous studies on *H. panicea* (*b* = 0.92; [46]) and other demosponge species, such as *Negombata magnifica* and *Suberites carnosus* [49, 50]. In *N. magnifica*, for instance, the specific respiration rate was found to be constant along the tested sponge size ranging from 10 to 60 g wet mass [49], yielded *b* = 1. Likewise, Reiswig [51] found that *b* = 1 in three tropical sponge species sometimes reaching a volume up to 2.5 L. Thus, while a range of scaling exponents may apply for sponges, their *b* values, including those obtained here, are generally ≥ 1 and well above the theoretical value of ¾ or 2/3, as is frequently observed in modular animals ([39], see review in [42]).

Methodological and biological factors may contribute to the variability of the scaling exponents in sponges. One possible cause for the deviation of our power exponent from those found in other studies is the low number of replicates, particularly within the large size group. Other, biological causes may be linked to differences in growth forms, metabolic states (i.e. the degree of activity) of sponges, or the variable contribution of sponge symbionts to overall sponge metabolism [42]. In addition, uncoordinated filter-feeding behaviour, as is observed in modules and specimens of *H. panicea* [52] or other sponge species [53], can affect their specific metabolic activity [22]. Such uncoordinated filter-feeding could explain metabolic scaling exponents of *b* < 1 in sponges, but it would likely not cause the *b* to exceed the value of 1.

Values of *b* > 1, however, have been observed in salps, prokaryotes and in the embryonic stages of animals including birds ([38] and references therein). In prokaryotes, the positive allometry results from the increase in metabolic capacity provided by the rising number of genes and enzymes in larger cells [54]. Thus, the positive allometric relationship determined in the present study could imply size-related changes in the metabolic demand in our *H. panicea* sponges [39, 46]. In any event, sponges in general, with our study included, are capable of compensating for the increased metabolic demands implied with larger size.

Linear allometry in sponges has been rationalized in a number of ways, including the view that sponges represent modular colonies of water-pumping choanocyte chambers, where the growth constitutes a proportional increase in the number of respiring cells [46]. Others have linked metabolic isometry in sponges with the homogeneous structure and porosity of the sponge interior, allowing them to overcome surface constraints of material exchange [49, 50]. Our experiments reinforce the recent hypothesis that isometric scaling in sponges results from their modular body architecture [55]. Thus, in this view, metabolic isometry in sponges is an emergent property of the iterative propagation of morphological units during growth. This type of growth is similar to colonial Bryozoans or scleractinian corals, where the modular design often allows for growth without functional constraints (e.g. [45, 56].). The addition of morphological-functional units of the same size maintains a constant surface area-to-volume ratio and relieves from mass as well as energetic constraints (e.g. see reviews by [38, 42, 47]).

However, the aquiferous modules in sponges do not morphologically or functionally resemble zooids in Bryozoans or Cnidarians. Sponge modules lack functional specialization and derive from “true growth” rather than incomplete asexual reproduction (i.e. budding), as is the major mechanism during morphogenesis of zooids in colonial Bryzoans and Cnidarians [25]. Yet, the modular organization of the water canal system in sponges may similarly influence how metabolic substrates and oxygen are acquired and distributed within the sponge body.

We show in *H. panicea* sponges that growth proceeds through the addition of aquiferous modules in proportion to sponge size (Fig. 3). This is because the water-pumping power generated by choanocyte chambers can only efficiently supply a certain module volume due to the rising frictional resistance associated with increasing canal length [9, 10, 57, 58]. Transport mechanics of the internal water canal system thus likely impose allometric constraints on the module size. Indeed, the pumping modules in the sponges we explored were approximately the same size (*V*_*module*_ ± 95% confidence intervals) of 1.59 ± 0.22 mL (Table 1) corresponding well with the experimentally determined mean *V*_*module*_ (± SD) of 1.08 ± 1.01 mL for *H. panicea* sponges investigated in previous studies (calculated from Table 3 in [57]). Indeed, the present scaling of *R* with the number of modules (*n*_*modules*_) (Fig. 4C) implies that modules in our multi-oscula *H. panicea* sponges have probably approached a size optimized for their functional capacities [9]. Overall, this suggests that the addition of modules conserved in size enables *H. panicea* demosponges to increase in biomass/volume far beyond constraints limiting the size of their component modules [45, 47, 59, 60].

The degree of physical and physiological integration between modules is likely to affect metabolic scaling of the whole-sponge organism [42]. This is exemplified in the non-sponge colonial ascidians (sea squirt) *Botryloides simodensis*, where the metabolic scaling relationship changes from *b* = 0.799 to near-isometry (*b* = 0.95) when mutual interactions between zooids (i.e. modules) cease due to disintegration of the shared transport system [61]. The metabolic scaling relationship found in the present study points toward a low degree of integration of modules in our multi-oscula *H. panicea* sponges; in other words, the modules exist as independent pumping units [9, 24, 62]. Indeed, multi-oscula *H. panicea* can arrest water pumping via the closure of oscula openings in some modules, while keeping others open and retaining their pumping activity [52, 53, 63, 64].

The presence of only one exhalant opening in some sponge species does not necessarily imply that their metabolic rate would scale differently with size compared to our investigated *H. panicea* sponges. Care must be taken to compare sponge species on the same level of organization since defining an aquiferous module by the presence of an osculum [24] cannot be applied to all demosponge species due to their various growth forms. Yet, some single-osculum sponges, such as the giant barrel sponge *Xestospongia muta* can grow up more than 452 L in volume [27], which can still efficiently be supplied by active water-pumping [65]. In these single-osculum, barrel-shaped sponges, such as *X. muta* and *Verongula gigantea*, several aquiferous modules drain into a large spongocoel, i.e. the atrium that possess a “pseudo-osculum” [55]. In analogy to our multi-oscula *H. panicea*, the metabolic rate of these “single-osculum multi-modular” [55] sponges may thus be the product of the number of the openings exhaling into the atrium and the individual module respiration. Therefore, the metabolic rate of these single-osculum sponges may also scale isometrically if their modules are independent pumping units of similar size and metabolic requirement.

### Nature of oxygen uptake kinetics

We found a Hill model generally suitable for describing the relationship between the dry weight-specific respiration rate (*R*_*DW*_, µmol O_2_ h^−1^ g (DW)^−1^) and declining dissolved oxygen (*DO,* % AS) (Additional file 1: Figures S2, S3) in our *H. panicea* sponges. For large specimens, the pattern of oxygen uptake during drawdown to anoxia displayed a hyperbolic form characterized by nearly constant respiration at oxygen levels from 100% to ~ 20% air saturation, which was followed by a linear decrease in oxygen uptake as the *DO* further diminished (Fig. 2, Additional file 1: Figures S2, S3). This was also evident in experiments with small specimens (e.g. sponge *ID*1 or *ID*2), but the shape of the curve was different at the lower boundaries of hypoxia, where their rate of oxygen uptake decreased asymptotically as the *DO* approached anoxia (Fig. 2B, Additional file 1: Figures S2, S3). The sigmoid character of these curves indicates a kinetic response other than Michaelis–Menten in our small *H. panicea* specimens.

Indeed, deviations from the conventional hyperbolic saturation curve have previously been noticed during oxygen drawdown experiments with bacteria (e.g. [66]) and blue mussels [67], and such deviations may occur in a large number of aquatic invertebrates [68]. The non-hyperbolic relation between oxygen consumption and oxygen can be explained, for instance, by variations in water-pumping behaviour as a function of oxygen levels [67] or an organism’s ability to switch to anaerobic pathways and/or to rest its aerobic metabolism as oxygen declines. Given the relatively prolonged exposure of small specimens to severely reduced oxygen levels (Fig. 2), it is likely that behavioural changes may have occurred in response to low levels of dissolved oxygen [69]. If this is true, then our observation that only experiments including (small) specimens with a low number of aquiferous modules (*n*_*modules*_ = 1–2) yielded sigmoid saturation curves might be related to the potential of modules within large, multi-oscula *H. panicea* sponges to respond asynchronously to changes in their environment [52].

To our knowledge, sponges lack the capacity for a fermentation-based metabolism that could explain the respiratory independence on ambient oxygen observed in our small sponge specimens. However, the asymptotical decrease in oxygen uptake at the lower boundaries of hypoxia implies a virtually resting aerobic metabolism in these sponges when oxygen becomes limiting, but this awaits future investigation. Metabolic quiescence has only been observed in freshwater sponge species, such as *Ephydatia muelleri*, who gain the capacity to withstand prolonged periods of anoxia in a gemmulated state [70]. Such dormant stages are not known from marine sponges and, therefore, would not have occurred in our experimental specimens. Indeed, some demosponges use systemic mechanisms to supply enough oxygen to their tissue when oxygen in the inspired seawater diminishes. The temperate sponge species *Suberites australiensis*, for instance, expands its aquiferous system at 5% AS, while its respiration rate remains unchanged [69]. Other demosponges, including *H. panicea* and *Geodia barretti*, reduce their ventilatory activity (= water-pumping rate) at oxygen levels as low as 4–7% AS [48, 71]. Thus, it is likely that the rate of water-pumping was severely reduced, maybe even arrested, in our sponges as the ambient oxygen declined to low concentrations.

Such a behavioural change was observed in recent oxygen drawdown experiments with small single-osculum explants of *H. panicea*, which seem to gradually reduce their pumping activity in response to the decreasing oxygen availability in the respiration chamber [23]. These were long-term experiments (several days), but if such behaviour also occurred in our short-term incubations, then the asymptotic pattern of oxygen uptake could reflect restructuring of the aquiferous system at low oxygen levels. Such modifications in response to hypoxia have previously been observed in other demosponges, such as *Polymastia crocea*, who presumably increase their surface-to-volume ratio for maximizing the diffusive oxygen uptake [69, 72].

### Respiratory oxygen dependency as a function of sponge size

**Fig. 6:**
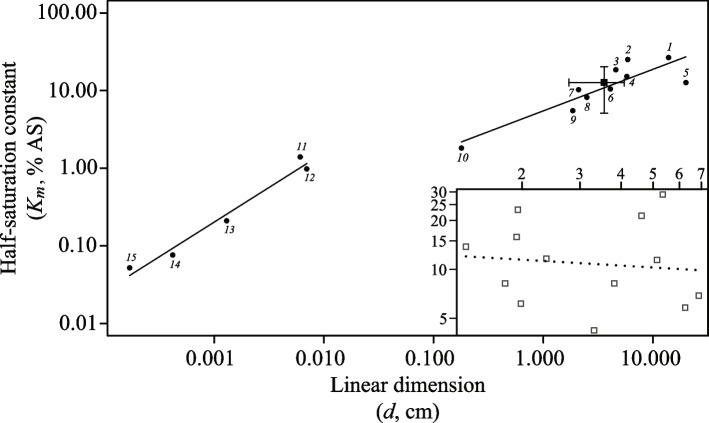
Compilation of half-saturation constants (*K*_*m*_, % AS) for respiration of various aerobic unicellular organisms and organelles (left) and multicellular organisms (right) as a function of their linear dimensions, plotted on a double logarithmic scale. Closed points represent data taken from ([2], and references therein), including crustaceans (1–4, 6, 8–9), fish (5,7), the annelid worm Tubifex (10), ciliates (11,12), the amoeba Acanthamoeba (13), yeast (14), and Tubifex mitochondria (15). Closed square shows mean *K*_*m*_ for several (*n* = 13) multi-oscula *H. panicea* sponges varying in size. Bars indicate standard deviations. Open squares in separate plot show *K*_*m*_ of *H. panicea* individuals investigated in the present study. Dotted regression line is shown

Oxygen concentrations can dictate the size of an aerobic organism. The internal transport of oxygen from the environment to sites (i.e. mitochondria) of oxidative metabolism in large organisms necessitates high ambient oxygen levels. Overall, one can envision the oxygen requirements of an organism through its *K*_*m*_ value [2], and for aerobic unicellular organisms, *K*_*m*_ scales positively with organism size (Fig. 6). This is also true for multicellular animals, but the scaling is different, where half-saturation values are lower than would be expected if the trend from single-celled organisms was followed (Fig. 6). The discontinuity between the trends for unicellular organisms and for multicellular animals occurs because the uptake and intracellular transport of oxygen in single-celled organisms is solely maintained by molecular diffusion. However, for multicellular animals exceeding a diameter of 1–2 mm, the internal oxygen requirements cannot be maintained by molecular diffusion alone. Thus, oxygen is supplied through vascular systems providing a more efficient oxygen transport and exchange than by diffusion alone. This more efficient oxygen exchange allows larger organisms at lower oxygen levels compared to the sizes possible from diffusion alone [2].

We have converted the size of our *Halichondria panicea* demosponges to mean diameter, allowing their data to be plotted with the other animals in Fig. 6. We see that *H. panicea* sponges fall within the range of other multicellular animals, such as crustaceans and fish (Fig. 6; *ID*1-10 from [2]). However, within *H. panicea* demosponges, there is no correlation between sponge size and their individual *K*_*m*_ values (Figs. 5 and 6). Thus, while *H. panicea* generally has *K*_*m*_ values compatible with their size when compared to other animals, there is no obvious impact of the size of an individual *H. panicea* on its *K*_*m*_ value. If we take the value of *K*_*m*_ to indicate the oxygen level at which the respiration and growth of an organism are significantly impacted, beyond a minimum threshold, the oxygen concentration itself would not seem to impact the size to which individuals of *H. panicea* can grow.

Our finding contrasts with previous studies on other solitary filter-feeders, such as bivalves, where *K*_*m*_ varies with the size of the animal [73–76]. In the blue mussel *Mytulis edulis*, for instance, the *K*_*m*_ increases with increasing body weight, indicating a higher respiratory dependency on ambient oxygen as the specimen grows larger ([76], but see [73, 75]). The lack of correlation between the *K*_*m*_ and sponge size from the present study may emerge from the modular architecture of the sponge body which grows by the repeated addition of morphological units including individual aquiferous systems (Fig. 3).

The type of growth in sponges, with the addition of individual, largely independent pumping units, is different from growth in other solitary organisms whose oxygen is supplied by a single network system, where an increase in size also increases the pathway length for the internal transport of oxygen from the surface into the core of the organism. In multi-modular sponges, however, partitioning of the sponge circulatory system into modules of a conserved size probably maintains a constant oxygen translocation distance across the sponge sizes. In this way, all parts of the sponge body remain in close contact to the inspired seawater [77, 78].

### Implications for modern hypoxia and animal evolution

Current models incorporating both natural and anthropogenic stressors, such as elevated temperature and nutrient runoff, predict further expansion of oxygen-minimum zones (OMZs) and coastal hypoxia [79–82]. The predicted expansion of hypoxia will probably have far-reaching impacts on ecosystems, where the detrimental effect of low oxygen results in biodiversity shifts and attendant losses in ecosystem functioning and services [83, 84]. Sponges, including their associated microbiomes, are ecologically vital for marine ecosystems in deep as well as shallow waters since they can considerably modulate benthic nutrient cycling [85–87] and mediate the transfer of dissolved organic matter to higher trophic levels via the “sponge loop” [88–91]. Consequently, a loss or displacement of sponge communities, including ‘key engineering’ species, due to human-induced ocean deoxygenation would be a serious threat to ecosystems populated by sponges [86, 90, 92].

The present study further supports previous hypotheses that sponges could be favored in future low-oxygen environments [69, 72, 93]. The potential to grow independent of reduced oxygen levels, at least to a certain point, may enable sponge populations to occupy habitats where declining oxygen has a significant impact on other sessile, solitary (i.e. non-modular) organisms’ ability to grow. In fact, natural populations of sponges, and corals, often appear in dense communities in areas where the reduced oxygen excludes species assemblages with a lower tolerance to hypoxia [94, 95]. For instance, some glass sponges, including *Vazella pourtalesii*, form monospecific sponge grounds on the continental Scotian shelf off Nova Scotia (Canada), where the ambient seawater is nutrient-rich, and warm, but with reduced oxygen concentrations of < 175 µM O_2_ [96]. Sponges are also persistent members of benthic communities in OMZ environments, such as the Peruvian OMZ [97], where oxygen can be reduced to ~ 3–8 µM. Furthermore, along the Union and Dellwoud seamounts in the Canadian northeast Ocean Pacific, highest densities of unidentified cold-water sponge and coral species are found within the core of the OMZ at oxygen concentration as low as 0.2 ml L^−1^ [95].

Overall, some sponges can indeed thrive in natural habitats including oxygen levels approaching those under which we have shown *H. panicea* sponges could potentially grow. The widespread tolerance to hypoxia across today’s sponge species (reviewed in [69]) could be an ancestral property of modern sponges given the simple sponge architecture and the likelihood that this simple architecture was established early in sponge evolution including basic characteristics such as particle capture and feeding [98], the choanocyte chamber [99] and the water canal system [100].

Sponges likely emerged early in animal evolution and likely represent the earliest evolved of the extant animal lineages [101, 102], with a history that may date back to 750 to 800 million years ago (e.g. [102]). It is also likely that they evolved in an environment with significantly lower oxygen levels than today, with levels that are uncertain, but with recent estimates suggesting large variability and concentrations ranging from between 1 to 50% of present levels [103, 104]. Given our results, one might expect that large sponges would be part of the geologic record back to their earliest evolution, even in a relatively low-oxygen world.

This is not the case. Putative sponge-like fossils can be found in rocks from the Tonian Period about 890 million years ago [105], the Cryogenian Period around 660 million years ago [106] and from the Ediacaran Period around 600 million years ago [107]. There are other reports of Neoproterozoic sponges as well (see [108]), but these fossils are not abundant, some are quite small [107] and they generally all lack the full set of features that would make definitive sponge interpretations [108]. Thus, despite the ability of sponges to grow to large size in a low-oxygen environment, definitive sponges, and sponges of large size are not observed until the early Cambrian Period [108–111].

Why, then, is the Precambrian record of sponges so sparse? This question is not new (e.g. [108, 112]) but our results, combined with other physiological studies on the low-oxygen tolerance of sponges [48, 113], suggest that oxygen availability was not likely the reason. Some have suggested that the lack of sponges could reflect a preservational bias, where the specifics of ocean and sediment chemistry mitigated against sponge preservation [112]. However, well-preserved fossils are found in Ediacaran-aged rocks [114], including the Ediacaran fauna that likely represent fossil animals. Thus, it is not so clear how a preservational bias might have selected against sponge preservation. Perhaps sponges had not evolved until the Cambrian Period. This explanation would seem to contradict molecular clock estimates for an earlier emergence of sponges and at least some fossil evidence for sponges themselves.

Thus, in one suggestion, Precambrian sponges may have been small and difficult to preserve; not because of size limitations on oxygen availability, but because of a later evolution of sponge modularity, meaning the ability of sponges to assemble individual aquiferous modules to generate a large sponge body. In fact, most living sponges possess a modularly designed aquiferous system, with multiple exhalant openings, while earliest known sponges (from the early Cambrian) might have been primarily solitary [115]. In this view, modular organization may be considered as an advanced state that presumably mediates more efficient filter-feeding [115–117] and persistence.

However, at least some fossil records exhibit characteristics of contemporary, adult sponges with apparent multiple oscula-like structures [107], suggesting multiple aquiferous modules [24, 118]. In the case of the early Ediacaran-aged fossil reported by Yin et al. [107], the aquiferous modules, if indeed this is what they were, are tiny. If these were indeed aquiferous modules, then sponge modularity evolved early but the (maximal) module size was considerably smaller than in Phanerozoic-aged sponges. If this is true, then large sponges probably evolved only when the arrangement of choanocyte chambers and water canals allowed for larger water-pumping units. Still, regardless of the reason limiting sponge size during early sponge evolution, our results suggest that it was not oxygen.

## Conclusions

The present study demonstrates that sponges scale differently with increasing size than other aerobic organisms where the large body size has an impact on the organisms’ metabolism and its dependency on ambient oxygen. We found that the respiration rate increased with increasing size of multi-oscula *H. panicea* sponges at a rate much faster than predicted from Kleiber’s law, but consistent with growth accompanied by the addition of independent aquiferous modules of similar size. We also provide evidence that low oxygen may not have an impact on the size to which *H. panicea* sponge individuals can eventually grow. Our findings indicate that the modular organization of the aquiferous system relaxes from surface area-to-volume ratio, mass and energetic constraints typically associated with individual growth. The addition of (independent) modules conserved in size may compensate for both the increased metabolic and the oxygen demands implied with larger size. We hypothesize that the progression of the sponge’ aquiferous system into a modular architecture may have contributed to the evolutionary success of sponges to colonize habitats when/where low oxygen concentrations have a significant impact on other sessile organisms’ ability to grow. More detailed knowledge on the evolution of sponge modularity could advance our understanding of the enigmatic fossil records of sponges and the emergence of large animals on Earth.

## Methods

### Sponge collection

Specimens of *Halichondria panicea* of varying size and number of oscula, i.e. modules, were collected from the inlet to Kerteminde Fjord, Fyn, Denmark and were transported to the laboratory in the Marine Biological Research Center (MRC) in Kerteminde. In the laboratory, specimens were thoroughly cleaned from epibiota and kept in a flow-through aquarium (30 L) with aerated seawater (~ 12–15°C, ~ 18–22 PSU) for less than 24 h prior to the start of respiration experiments.

### Respiration rate measurements

We explored the sponge respiration rate (*R*, µmol O_2_ h^−1^) as a function of five different size parameters, i.e. the number of modules (*n*_*modules*_), dry weight (*DW*, g), volume (*V*_*sp*_, mL), module volume (*V*_*module*_, mL) and estimated spherical diameter (*d*, cm) in 13 sponge specimens. The respiration rate was determined as the draw-down rate of dissolved oxygen (*DO*; % air saturation) by a sponge placed in tightly closed respiration chambers filled initially with fully oxygenated and well-stirred 0.2 µm sterile-filtered seawater (20 PSU). Respiration chambers with and without (control) sponges were submerged in a temperature-controlled water-bath to maintain a stable temperature (12.4 ± 0.1 °C) throughout all experiments (Fig. 1). The experimental setup was covered to eliminate light exposure, thus limiting the oxygen production of potential photosynthetic organisms in the sponge interior [119, 120] and in the respiration chambers.

The dissolved oxygen concentrations (*DO*) in both the experimental and control chambers were recorded with a FireStingO_2_ optical oxygen meter (PyroScience) using Pyro Oxygen Logger^©^ software. Oxygen sensors were calibrated against well-oxygenated (100% air-saturation, AS) as well as deoxygenated (by adding sodium dithionite) sterile-filtered seawater water (0% AS). The *DO* measurements were recorded at intervals ranging from 1–180 s and values were temperature corrected by the Pyro Oxygen Logger^©^ software. The experiments were terminated a few hours after reaching anoxia, and they lasted between 13–44 h, depending on the relationship between sponge respiration rate and respiration chamber size. Data were smoothed by calculating the running average over *n* datapoints (n = 15 to 1799 datapoints, depending on the timespan between two observations and the scattering of the data, see Additional file 1: Table S1).

The *DO* (% AS) was converted to µmol O_2_ L^−1^ using the fully saturated oxygen concentrations in saline water (20 PSU, 12 °C; cf. Table 6 in [121]) to calculate the respiration rate from the decrease of *DO* (μmol O_2_ L^−1^) over time, using the following equation [23]:

2 $$R = a \times V$$

where *R* is the respiration rate in µmol O_2_ h^−1^, *a* is the slope of the linear regression model (*LM*) for the decrease in *DO* in µmol O_2_ L^−1^ over time in hours for each time interval (see Additional file 1: Table S1) and *V* is the volume of water in the experimental chamber (*V* = 0.12—1.14 L). Respiration rates of the sponge specimens were standardized to dry weight *DW* (g) to determine the dry weight-specific respiration rate (*R*_*DW*,_ µmol O_2_ h^−1^ g (DW)^−1^)*.*

After each experiment, the number of modules (*n*_*modules*_) was determined as the number of oscula counted on the sponge. The water-displacement of the sponge volume (*V*_*sp*_), as well as the sponge wet weight (*WW*) and *DW* (100°C for 24h), were estimated according to Thomassen and Riisgård [46]. The volume per module was calculated as *V*_*sp*_*/n*_*modules*_ for each specimen. We also calculated the sponges’ diameter (*d,* cm) from *V*_*sp*_ (see Eq. 3) using a sphere as an approximation for the geometry of the sponge, and assuming that the surface area-to-volume ratio (*SV*-ratio) of a sphere represents well the optimized *SV*-ratio of a “porous” sponge body.

3 $$d = 2 \times \sqrt[3]{\frac{3V_{sp}}{{4}\pi}}$$

### Determining respiration kinetics of *Halichondria panicea*

Data were analyzed in R-studio (Version 1.1.442; [122]). For each experiment, we plotted the *R*_*DW*_ as a function of dissolved oxygen (*DO*, % AS) to estimate the maximum respiration rate (*R*_*max*_) and the O_2_ concentration providing half of *R*_*max*_, known as the half-saturation constant *K*_*m*_. Due to the various shapes of the saturation curves ranging from hyperbolic to sigmoid, we chose to fit a “Hill” model [123, 124] using a nonlinear least squares (nls) function from the R-package “Analysis of Dose–Response Curves v.3.0–1” [125]. The Hill model is expressed as:

4 $${R}_{DW}=\frac{{R}_{max}\times {DO}^{n}}{{K}_{m}^{n}+{DO}^{n}}$$

where *R*_*DW*_ is the dry weight-specific respiration rate, *R*_*max*_ is the maximum *R*_*DW*_, *DO* is the dissolved oxygen, *K*_*m*_ is the half-saturation constant, and *n* is the Hill coefficient. The *n* = 1 indicates a hyperbolic saturation curve for the *R*_*DW*_ as a function of the *DO*. In this case, the Hill equation is reduced to the Michaelis–Menten equation [126]. We recognize that the Hill equation can analyze functions of a different form than given by the traditional Michaelis–Menten equation, but the *K*_*m*_ value still represents the oxygen level at half-maximum respiration rate, so we will still refer to these Hill-equation-derived values as *K*_*m*_*.*

Outliers, i.e. initial fluctuations of *R* during some of our respiration rate measurements (e.g. *ID*3), were determined from diagnostic plots using the R-package “Tools for Nonlinear Regression Analysis” [127] and were excluded from further analyses. We defined outliers as deviations of the predicted Hill model, which had standardized residuals of the fitted model >  ± 2. Excluding these outliers from our analyses, however, had a minor effect on estimates of *K*_*m*_ and *R*_*max*_ for most of the specimens (see Additional file 1: Figure S3 in Supplementary Materials).

### Scaling of respiration rates with sponge size

The respiration rates (*R*_*max*_, µmol O_2_ h^−1^) determined from our oxygen drawdown experiments were plotted against the sponge dry-weight (*DW*, g) on a double logarithmic scale and fitted to the allometric equation (Eq. 1, [32]). Data were log_10_ transformed, and a linear regression was performed to estimate the scaling components (cf [65].).

Our initial hypothesis was that the metabolic rate in our multi-oscula *H. panicea* sponges would increase in direct proportion to sponge size (e.g. [46].), i.e. with a *b* = 1 in Eq. 1. To test this hypothesis, we performed a two-sided *t*-test to determine if the metabolic relationship revealed from our data was significantly different from our hypothesized isometry with *b* = 1 [128, 129]. For this, we tested against a randomly generated dataset that followed a scaling relationship with the power exponent of *b* = 1, using the same size range and intercept as determined for our empirical dataset.

### Supplementary Information

**Additional file 1: Table S1.** Modelling and statistical results of the Hill-model fitted for respiration rates of several *H. panicea* sponges as a function of oxygen levels. **Fig. S1.** Oxygen drawdown over time measured in respiration chamber with and without a sponge. **Fig. S2.** Dry weight-specific respiration rate *R*_*DW*_ as a function of dissolved oxygen (*DO*) for several *H. panicea* sponges. Outliers not considered for determinations of the maximum respiration rate *R*_*max*_ and the half-saturation constant *K*_*m*_ are shown. **Fig. S3.** Dry weight-specific respiration rate *R*_*DW*_ as a function of dissolved oxygen (*DO*) for several *H. panicea* sponges.

## Data Availability

The datasets generated and/or analysed during the current study are available from the corresponding author on reasonable request.
