# Supplementary material for: Respiration kinetics and allometric scaling in the demosponge Halichondria panicea
Source: BMC Ecol Evol. 2023 Sep 19;23:53. doi: 10.1186/s12862-023-02163-5 (PMC10507823; doi:10.1186/s12862-023-02163-5)
Supplement: Supplementary file 1 — Additional file 1: Table S1. Modelling and statistical results of the Hill-model fitted for respiration rates of several H. panicea sponges as a function of oxygen levels. Fig. S1. Oxygen drawdown over time measured in respiration chamber with and without a sponge. Fig. S2. Dry weight-specific respiration rate RDW as a function of dissolved oxygen (DO) for several H. panicea sponges. Outliers not considered for determinations of the maximum respiration rate Rmax and the half-saturation constant Km are shown. Fig. S3. Dry weight-specific respiration rate RDW as a function of dissolved oxygen (DO) for several H. panicea sponges. [file 12862_2023_2163_MOESM1_ESM.pdf]

The following supplement accompanies the article

Respiration kinetics and allometric scaling in the demosponge *Halichondria panicea*

Lars Kumala[1,2,3]*, Malte Thomsen[1,2,3], and Donald E. Canfield[1,3,4]

[1] *Department of Biology, University of Southern Denmark, 5230 Odense M, Denmark*

[2] *Marine Biological Research Centre, University of Southern Denmark, DK-5300 Kerteminde, Denmark*

[3]*Nordcee, Department of Biology, University of Southern Denmark, 5230 Odense M, Denmark*

[4]*Danish Institute for Advanced Study (DIAS), University of Southern Denmark, 5230 Odense M, Denmark*

**Correspondence: Lars Kumala, E-mail: kumala@biology.sdu.dk*

Table S1 Modelling and statistical results of the Hill-model fitted for respiration rates of several *H. panicea* sponges (*ID*1-13) as a function of oxygen levels (Fig. S2). Sample interval (*t*, s), datapoints used for smoothing with running average (*RAvg*), time intervals used to calculate respiration rates using linear regressions (t_{Reg}, s), the goodness of the fit represented by R^2, and residual standard error (*RSE*).

ID	*t* (s)	*RAvg*	t_{Reg} (s)	R^2	*RSE*
1	10	599	1200	0.999	0.199
2	10	599	1800	0.991	0.602
3	180	29	1080	0.995	0.433
4	10	899	1800	0.995	0.246
5	180	29	1800	0.994	0.230
6	180	29	1080	0.996	0.187
7	60	15	180	0.996	0.173
8	180	29	540	0.999	0.114
9	1	349	360	0.997	0.354
10	1	1199	800	0.982	1.192
11	1	599	1200	0.988	1.651
12	1	1799	500	0.991	0.348
13	1	179	180	0.998	0.117

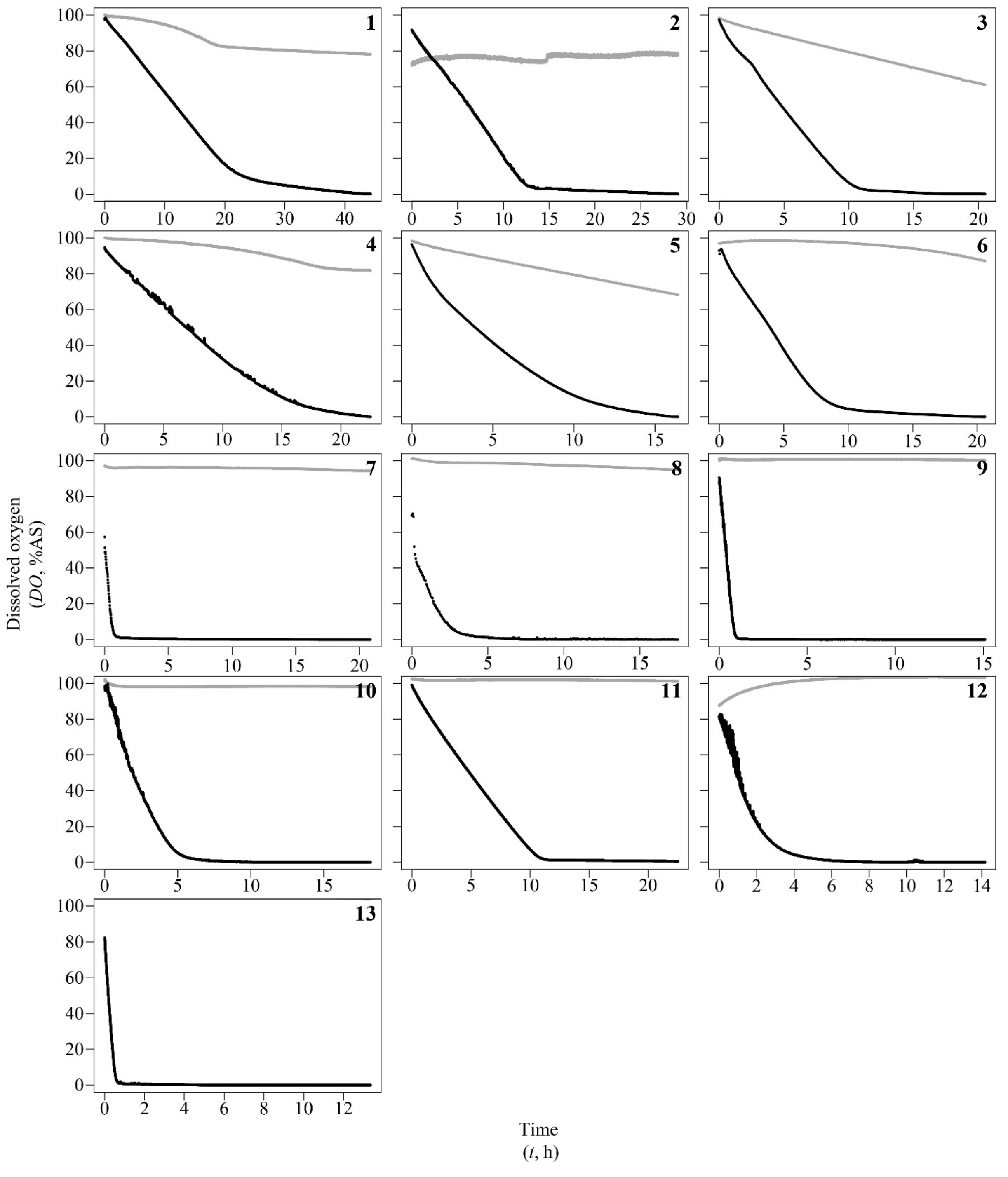

Fig. S1 Measured oxygen (*DO*, % AS) drawdown over time (h) in respiration chamber with (*ID*1-13, dark line) and without a sponge (= control, grey line).

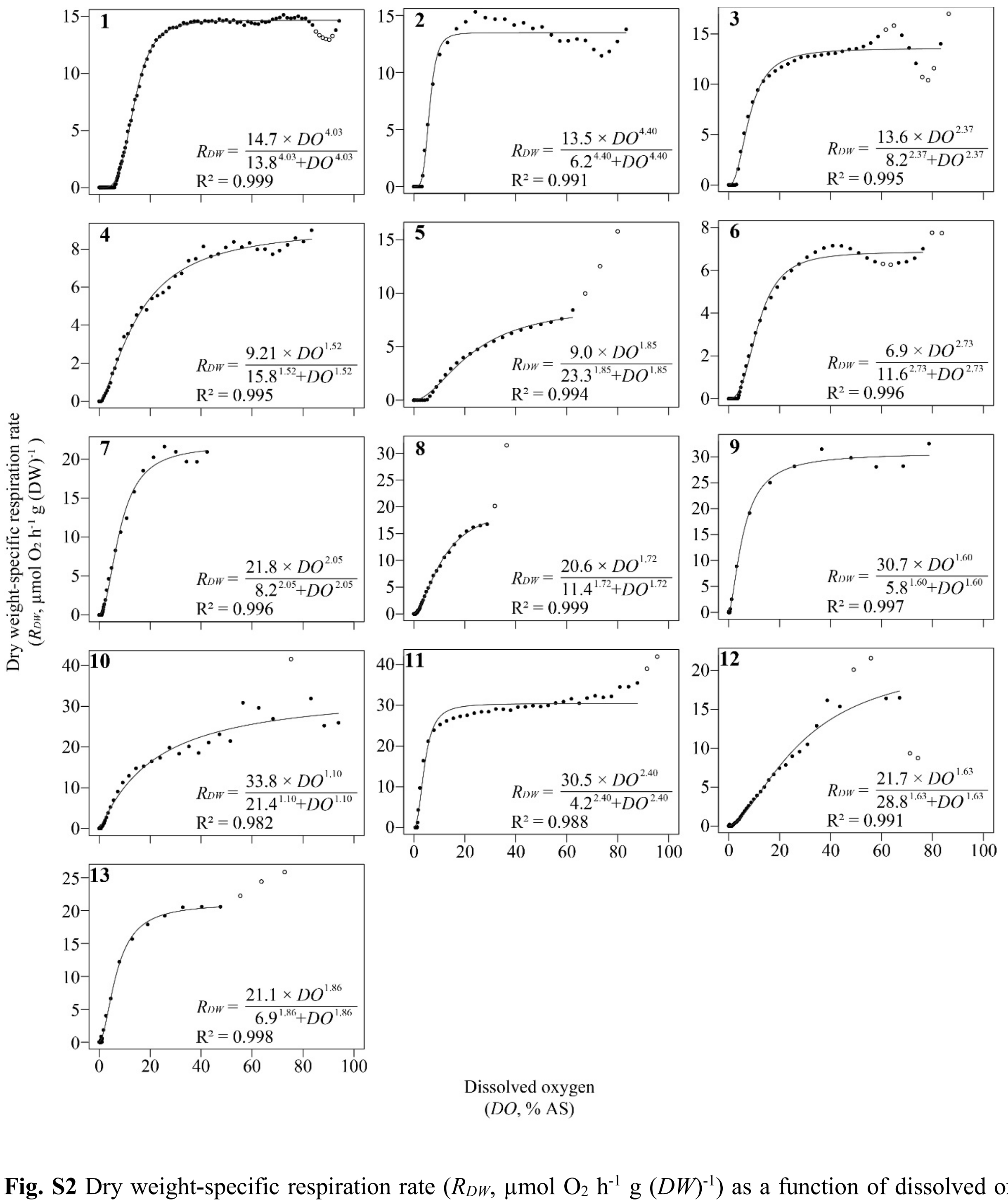

Fig. S2 Dry weight-specific respiration rate (R_{DW}, µmol O_2 h^{-1} g $(DW)^{-1}$) as a function of dissolved oxygen (*DO,* % AS) for several *H. panicea* sponges (*ID*1-13). Closed circles show the sponge' respiration rate, open circles represent outliers, i.e. initial fluctuations in the measurements of the R_{DW}. Outliers were not considered for determinations of the maximum respiration rate ($R_{\max}$) and the half-saturation constant (K_m). Dark line indicates the fitted 'Hill-model', and its equation is shown.

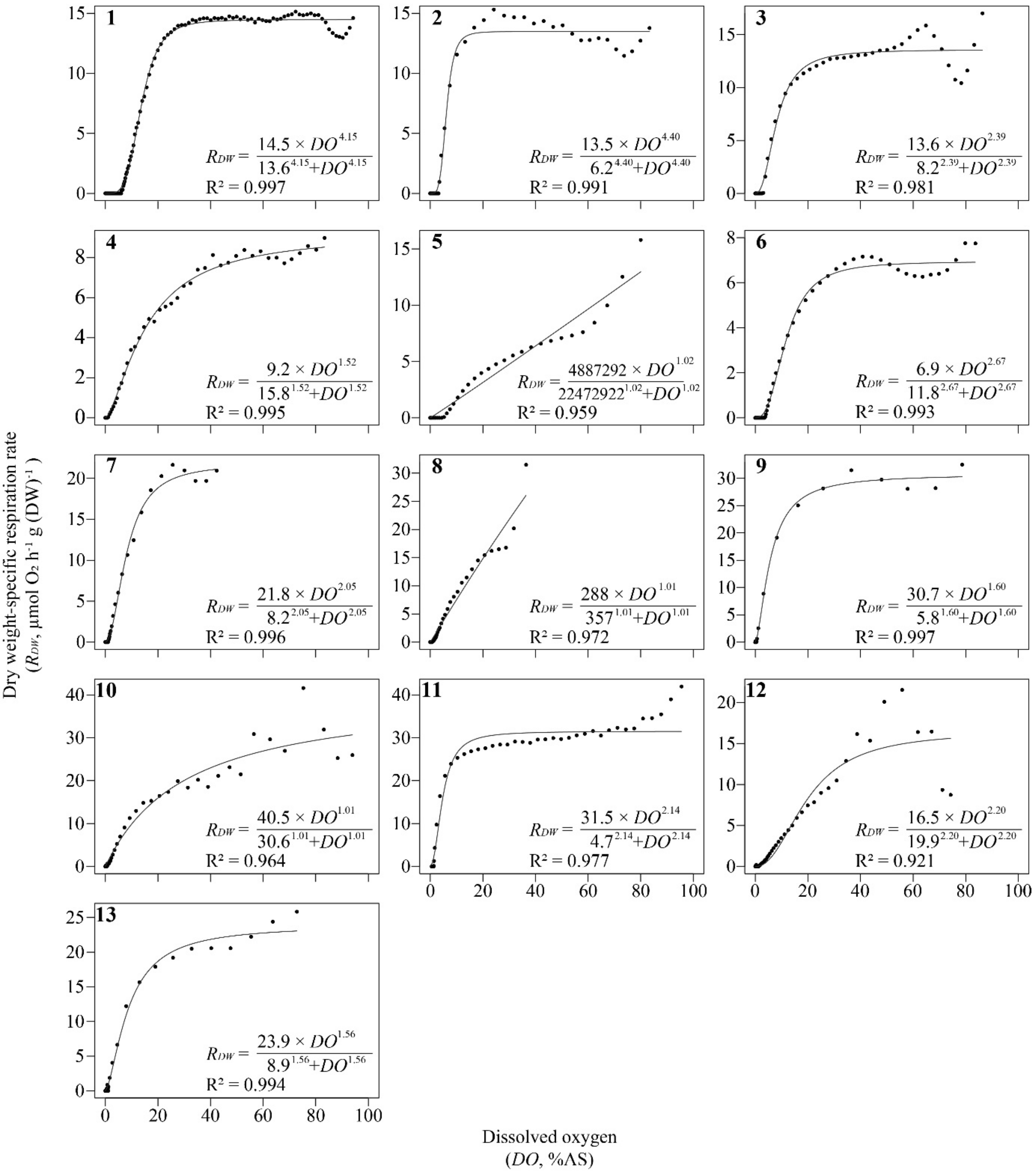

Fig. S3 Dry weight-specific respiration rate (R_{DW}, µmol O$_2$ h^{-1} g $(DW)^{-1}$) as a function of dissolved oxygen (*DO,* % AS) for several *H. panicea* sponges (*ID*1-13). Closed circles show the sponge' respiration rate. Outliers not considered for determinations of the maximum respiration rate (R_{max}) and the half-saturation constant K_m, are shown in Fig. S2. Dark line shows the fitted 'Hill-model', and its equation is shown.
